# *MTHFR* and risk of stroke and heart disease in a low-folate population: a prospective study of 156 000 Chinese adults

**DOI:** 10.1093/ije/dyad147

**Published:** 2023-10-28

**Authors:** Derrick A Bennett, Sarah Parish, Iona Y Millwood, Yu Guo, Yiping Chen, Iain Turnbull, Ling Yang, Jun Lv, Canqing Yu, George Davey Smith, Yongjun Wang, Yilong Wang, Richard Peto, Rory Collins, Robin G Walters, Liming Li, Zhengming Chen, Robert Clarke, the China Kadoorie Biobank (CKB) Study Collaborative Group, Junshi Chen, Zhengming Chen, Robert Clarke, Rory Collins, Liming Li, Chen Wang, Jun Lv, Richard Peto, Robin Walters, Daniel Avery, Maxim Barnard, Derrick Bennett, Ruth Boxall, Ka Hung Chan, Yiping Chen, Zhengming Chen, Johnathan Clarke, Robert Clarke, Huaidong Du, Ahmed Edris Mohamed, Hannah Fry, Simon Gilbert, Pek Kei Im, Andri Iona, Maria Kakkoura, Christiana Kartsonaki, Hubert Lam, Kuang Lin, James Liu, Mohsen Mazidi, Iona Millwood, Sam Morris, Qunhua Nie, Alfred Pozarickij, Paul Ryder, Saredo Said, Dan Schmidt, Becky Stevens, Iain Turnbull, Robin Walters, Baihan Wang, Lin Wang, Neil Wright, Ling Yang, Xiaoming Yang, Pang Yao, Xiao Han, Can Hou, Qingmei Xia, Chao Liu, Jun Lv, Pei Pei, Dianjianyi Sun, Canqing Yu Naying Chen, Duo Liu, Zhenzhu Tang Ningyu Chen, Qilian Jiang, Jian Lan, Mingqiang Li, Yun Liu, Fanwen Meng, Jinhuai Meng, Rong Pan, Yulu Qin, Ping Wang, Sisi Wang, Liuping Wei, Liyuan Zhou Caixia Dong, Pengfei Ge, Xiaolan Ren Zhongxiao Li, Enke Mao, Tao Wang, Hui Zhang, Xi Zhang, Jinyan Chen, Ximin Hu, Xiaohuan Wang Zhendong Guo, Huimei Li, Yilei Li, Min Weng, Shukuan Wu Shichun Yan, Mingyuan Zou, Xue Zhou Ziyan Guo, Quan Kang, Yanjie Li, Bo Yu, Qinai Xu Liang Chang, Lei Fan, Shixian Feng, Ding Zhang, Gang Zhou Yulian Gao, Tianyou He, Pan He, Chen Hu, Huarong Sun, Xukui Zhang Biyun Chen, Zhongxi Fu, Yuelong Huang, Huilin Liu, Qiaohua Xu, Li Yin Huajun Long, Xin Xu, Hao Zhang, Libo Zhang, Jian Su, Ran Tao, Ming Wu, Jie Yang, Jinyi Zhou, Yonglin Zhou Yihe Hu, Yujie Hua, Jianrong Jin, Fang Liu, Jingchao Liu, Yan Lu, Liangcai Ma, Aiyu Tang, Jun Zhang, Liang Cheng, Ranran Du, Ruqin Gao, Feifei Li, Shanpeng Li, Yongmei Liu, Feng Ning, Zengchang Pang, Xiaohui Sun, Xiaocao Tian, Shaojie Wang, Yaoming Zhai, Hua Zhang, Wei Hou, Silu Lv, Junzheng Wang, Xiaofang Chen, Xianping Wu, Ningmei Zhang, Xiaoyu Chang, Xiaofang Chen, Jianguo Li, Jiaqiu Liu, Guojin Luo, Qiang Sun, Xunfu Zhong, Weiwei Gong, Ruying Hu, Hao Wang, Meng Wang, Min Yu, Lingli Chen, Qijun Gu, Dongxia Pan, Chunmei Wang, Kaixu Xie, Xiaoyi Zhang

**Affiliations:** Clinical Trial Service Unit, Nuffield Department of Population Health, University of Oxford, Oxford, UK; Clinical Trial Service Unit, Nuffield Department of Population Health, University of Oxford, Oxford, UK; Medical Research Council Health Research Unit, Nuffield Department of Population Health, University of Oxford, Oxford, UK; Clinical Trial Service Unit, Nuffield Department of Population Health, University of Oxford, Oxford, UK; Fuwai Hospital Chinese Academy of Medical Sciences, National Center for Cardiovascular Diseases, Beijing, China; Clinical Trial Service Unit, Nuffield Department of Population Health, University of Oxford, Oxford, UK; Medical Research Council Health Research Unit, Nuffield Department of Population Health, University of Oxford, Oxford, UK; Clinical Trial Service Unit, Nuffield Department of Population Health, University of Oxford, Oxford, UK; Clinical Trial Service Unit, Nuffield Department of Population Health, University of Oxford, Oxford, UK; Medical Research Council Health Research Unit, Nuffield Department of Population Health, University of Oxford, Oxford, UK; Department of Epidemiology and Biostatistics, School of Public Health, Peking University Health Science Center, Beijing, China; Peking University Center for Public Health and Epidemic Preparedness and Response, Beijing, China; Department of Epidemiology and Biostatistics, School of Public Health, Peking University Health Science Center, Beijing, China; Peking University Center for Public Health and Epidemic Preparedness and Response, Beijing, China; MRC Integrative Epidemiology Unit, University of Bristol, Bristol, UK; China National Clinical Research Center for Neurological Diseases, Beijing Tiantan Hospital, Capital Medical University, Beijing, China; Department of Neurology, Beijing Tiantan Hospital, Capital Medical University, Beijing, China; China National Clinical Research Center for Neurological Diseases, Beijing Tiantan Hospital, Capital Medical University, Beijing, China; Department of Neurology, Beijing Tiantan Hospital, Capital Medical University, Beijing, China; Clinical Trial Service Unit, Nuffield Department of Population Health, University of Oxford, Oxford, UK; Clinical Trial Service Unit, Nuffield Department of Population Health, University of Oxford, Oxford, UK; Clinical Trial Service Unit, Nuffield Department of Population Health, University of Oxford, Oxford, UK; Medical Research Council Health Research Unit, Nuffield Department of Population Health, University of Oxford, Oxford, UK; Department of Epidemiology and Biostatistics, School of Public Health, Peking University Health Science Center, Beijing, China; Peking University Center for Public Health and Epidemic Preparedness and Response, Beijing, China; Clinical Trial Service Unit, Nuffield Department of Population Health, University of Oxford, Oxford, UK; Clinical Trial Service Unit, Nuffield Department of Population Health, University of Oxford, Oxford, UK

**Keywords:** *MTHFR*, folate, homocysteine, stroke, Mendelian randomization, China

## Abstract

**Background:**

The relevance of folic acid for stroke prevention in low-folate populations such as in China is uncertain. Genetic studies of the *methylenetetrahydrofolate reductase* (*MTHFR*) C677T polymorphism, which increases plasma homocysteine (tHcy) levels, could clarify the causal relevance of elevated tHcy levels for stroke, ischaemic heart disease (IHD) and other diseases in populations without folic acid fortification.

**Methods:**

In the prospective China Kadoorie Biobank, 156 253 participants were genotyped for *MTHFR* and 12 240 developed a stroke during the 12-year follow-up. Logistic regression was used to estimate region-specific odds ratios (ORs) for total stroke and stroke types, IHD and other diseases comparing TT genotype for *MTHFR* C677T (two thymine alleles at position 677 of *MTHFR* C677T polymorphism) vs CC (two cytosine alleles) after adjustment for age and sex, and these were combined using inverse-variance weighting.

**Results:**

Overall, 21% of participants had TT genotypes, but this varied from 5% to 41% across the 10 study regions. Individuals with TT genotypes had 13% (adjusted OR 1.13, 95% CI 1.09–1.17) higher risks of any stroke [with a 2-fold stronger association with intracerebral haemorrhage (1.24, 1.17–1.32) than for ischaemic stroke (1.11, 1.07–1.15)] than the reference CC genotype. In contrast, *MTHFR* C677T was unrelated to risk of IHD or any other non-vascular diseases, including cancer, diabetes and chronic obstructive lung disease.

**Conclusions:**

In Chinese adults, the *MTHFR* C677T polymorphism was associated with higher risks of stroke. The findings warrant corroboration by further trials of folic acid and implementation of mandatory folic acid fortification programmes for stroke prevention in low-folate populations.

Key Messages

- Individuals with the TT genotype for *MTHFR* C677T (two thymine alleles at position 677 of *MTHFR* C677T polymorphism), which occurs in ∼10% in Western populations, have ∼25% (∼3 µmol/L) higher plasma homocysteine (tHcy) than those with the CC genotype (two cytosine alleles) in populations without folic acid fortification, but the effects are almost fully attenuated by effective folic acid fortification.
- Variable implementation of folic acid fortification worldwide has resulted in 2- to 4-fold differences in mean plasma folate concentrations between countries.
- In a prospective study of 156 253 participants in the China Kadoorie Biobank, ∼21% had TT homozygotes for *MTHFR* C677T. TT homozygotes had 13% higher risks of all strokes (24% higher risks of intracerebral haemorrhage and 11% higher risks of ischaemic stroke) than CC homozygotes.
- *MTHFR* C677T was unrelated to ischaemic heart disease or to any of the other non-vascular diseases studied.
- The findings should prompt further trials of folic acid supplementation for stroke prevention in China and implementation of effective mandatory folic acid fortification programmes for stroke prevention.

## Introduction

Homocysteine (tHcy) is a sulphur-containing amino acid that plays a key role in one-carbon metabolism (Supplementary Figure S1, available as Supplementary data at *IJE* online) and genetic defects or deficiency of folic acid or vitamin B12 cause elevated plasma tHcy levels.^1^ Elevated plasma tHcy levels have been associated with higher risks of cardiovascular disease (CVD)^2^^,^^3^ and some non-CVD outcomes,^4^ but the causal relevance of these associations is uncertain and may differ importantly between populations with or without folic acid fortification.

Methylenetetrahydrofolate reductase (MTHFR) is an enzyme that controls a key branch point in folate metabolism and regulates the supply of one-carbon units required for protein synthesis. MTHFR uses folate to metabolize and thereby remove tHcy (Supplementary Figure S1, available as Supplementary data at *IJE* onlinevv). A common genetic variant *MTHFR* C677T (rs1801133) (T allele frequency ∼10% in European ancestry populations) greatly reduces enzyme efficiency. TT genotypes for this polymorphism (i.e. two thymine alleles at position 677 of *MTHFR* C677T) have ∼25% (∼3 µmol/L) higher plasma tHcy concentrations than the reference genotype (denoted as CC as they have two cytosine alleles).^5^^,^^6^ However, the tHcy-elevating effects of TT homozygotes for *MTHFR* C677T on plasma tHcy concentrations are substantially attenuated by folic acid fortification.

Folic acid fortification has been widely implemented worldwide over the last three decades and it has reduced the incidence of neural tube defects,^7–9^ but variable implementation of different types of folic acid fortification has resulted in substantial differences in mean plasma folate levels in populations with mandatory, voluntary or no folic acid fortification (30–40 vs 20–25 vs 10–15 nmol/L, respectively).^10–12^ The UK government has recently signalled its intention to change from voluntary to mandatory folic acid fortification but many other countries, including China, have not yet implemented any effective food fortification with folic acid.^10^

Mendelian randomization (MR) studies have reported positive associations of TT vs CC homozygotes for *MTHFR* C677T with total stroke in non-fortified populations, but not in populations with folic acid fortification.^6^ The *MTHFR* TT frequency also varies substantially between populations (5–40%) and hence many studies of *MTHFR* C677T have been constrained by incomplete control for population stratification. Randomized trials have reported no beneficial effects of folic acid supplementation on stroke in Western populations but the Chinese Stroke Primary Prevention Trial (CSPPT) reported that folic acid supplementation lowered risk of total stroke by 21%.^14^ The discrepant results of the folic acid trials^13^^,^^14^ and MR studies^15–17^ between Chinese and Western populations may reflect the differences in folic acid fortification policies.

The aims of this study were to: (i) assess the associations of the *MTHFR C677T* polymorphism with total stroke, including ischaemic stroke (IS) and intracerebral haemorrhage (ICH) types, and with ischaemic heart disease (IHD), including myocardial infarction (MI), and other IHD types after stratifying for the 10 study regions and adjusting for principal components; and (ii) examine the associations of the *MTHFR C677T* polymorphism with site-specific cancers and other diseases previously linked with low-folate status in a large prospective Chinese cohort study.

## Methods

### Study population

Details of the design, survey methods, procedures and participant characteristics of the China Kadoorie Biobank (CKB) study have been previously reported.^18^^,^^19^ In brief, the CKB is a prospective cohort study of 512 715 adults who were recruited between 25 June 2004 and 15 July 2008 from 10 diverse rural and urban regions of China (Supplementary Methods, available as Supplementary data at *IJE* online).

### Follow-up for disease outcomes

Information about incident disease and cause-specific mortality was obtained by electronic linkage, via the unique national identification number, to established local mortality (cause-specific) and morbidity (for stroke, IHD, cancer and diabetes) registries and to the health insurance system that records any hospitalization episodes and procedures.^20^^,^^21^ All disease diagnoses were coded using the Tenth International Classification of Diseases (ICD-10), blinded to any baseline information.

### Major disease outcomes

For the present study, the main vascular disease outcomes included first non-fatal or fatal IS, ICH, total stroke, acute MI, other acute IHD and all IHD (Supplementary Table S1, available as Supplementary data at *IJE* online). Stroke types were classified into IS, ICH and other pathologic types.^20^^,^^21^ The main non-vascular disease outcomes included total incident cancer and cancers of the colon, lung, stomach and breast. Other non-vascular diseases included diseases previously reported to be associated with elevated tHcy levels (Supplementary Table S1, available as Supplementary data at *IJE* online) in addition to a phenome-wide analysis of 41 major disease groups.

### Genotyping procedures

Genotyping of the *MTHFR C677T* single-nucleotide polymorphism (SNP) was performed at BGI Genomics Company, Shenzhen, China by genotyping 384 candidate SNPs using Illumina Golden Gate technology (Illumina, San Diego) in 92 987 randomly selected participants. In addition, genome-wide genotyping using a custom-designed Affymetrix 800K SNP arrays (including *MTHFR C677T*) was performed in 75 982 participants who were randomly selected from the CKB population and an additional 24 658 CKB participants were selected for nested case–control studies of incident cardiovascular or respiratory diseases.

After excluding a small overlap, the present analyses involved 162 366 participants with genotyping data for *MTHFR C677T* (rs1801133), of whom 151 393 were randomly selected participants and 10 973 were selected because they had a stroke or IHD event during follow-up (Supplementary Figure S2, available as Supplementary data at *IJE* online). After exclusion of 6133 participants with non-local ancestry or missing data for regional principal components (PCs), a total of 156 253 participants were included in the final analyses (Supplementary Figure S2, available as Supplementary data at *IJE* online).

For all vascular disease outcomes, a set of common controls were used that excluded individuals with a prior history of IHD, stroke or transient ischaemic attack, incident IHD or incident major vascular events (fatal or non-fatal MI, fatal or non-fatal stroke or death from cardiovascular disease: Supplementary Figure S2, available as Supplementary data at *IJE* online). For non-vascular disease outcomes, we used controls that separately excluded individuals with any of the specific non-vascular diseases for each comparison.

### Statistical methods

Baseline characteristics were analysed for the overall CKB cohort and the genotyped subset. The *MTHFR* C677T genotype was assessed for departure from Hardy–Weinberg Equilibrium (HWE) by region and overall using a HWE chi-square test. Principal component analysis identified 11 PCs informative for CKB population structure (Supplementary Methods, available as Supplementary data at *IJE* online).^22^ The associations of *MTHFR* C677T genotypes with continuous traits were assessed by using linear regression after stratification by region and adjustment for age, sex and regional PCs. Binary traits were stratified by region; adjusted for age, sex and regional PCs; and presented as adjusted prevalence.

Logistic regression was used to estimate the odds ratios (ORs) comparing TT vs CC genotypes of the *MTHFR* C677T for each disease outcome after stratification by region and adjusting for age, sex and regional PCs. The region-specific risk estimates were meta-analysed using inverse-variance weighting to yield overall ORs for disease outcomes, with the statistical importance of the effect sizes assessed using a likelihood ratio (LRT) chi-square statistic.

Additional analyses undertaken included: (i) using tHcy weighted analyses to obtain the effect of a 25% MTHFR-derived higher plasma tHcy concentration; and (ii) estimating effects per T allele for *MTHFR* C677T. Further analyses included stepwise comparisons of incremental adjustments in the logistic regression models by: (i) age, sex, region and regional PCs;^22^ (ii) additional adjustments for potential pleiotropic factors and (iii) other CVD risk factors to account for confounding (Supplementary Methods, available as Supplementary data at *IJE* online). Subgroup analyses were performed to assess potential effect modification for the main stroke outcomes by CVD risk factors and by genetic variants influencing alcohol consumption.^23^^,^^24^ Sensitivity analyses assessed the robustness of the main results after excluding prior disease in both cases and controls, or in addition after restricting analyses to population subsets with genome-wide association study data. All statistical analyses were conducted in SAS 9.4.

## Results

The baseline characteristics of the genotyped subset were similar to those of the overall CKB population (Supplementary Table S2, available as Supplementary data at *IJE* online). Among control participants, the prevalence of *MTHFR* C677T genotypes did not differ by age, sex, education, lifestyle factors, systolic blood pressure (SBP) or by measures of adiposity (Table 1). However, there were associations of *MTHFR* C677T with mean levels of blood pressure indices (diastolic blood pressure (DBP), pulse pressure and mean arterial pressure), height, weight and random blood glucose concentrations, but the absolute differences were modest. The associations of *MTHFR* C677T with blood pressure indices provide evidence for some pleiotropic effects on blood pressure (Table 1). Consistently with the findings in controls only, similar associations of *MTHFR* C677T with blood pressure types were observed for all participants (Supplementary Table S3, available as Supplementary data at *IJE* online).

**Table 1.** Associations of *MTHFR* C677T genotypes with control participant characteristics in China Kadoorie Biobank^a^

Participant characteristics	*MTHFR* genotypes in controls			*P* for trend
	CC (*n* = 38 908)	CT (*n* = 51 479)	TT (*n* = 23 185)	
Age, years^b^	50.0 (10.3)	50.1 (9.9)	50.0 (10.3)	0.52
Female^c^	60.3%	60.4%	59.7%	0.26
High school education or above^c^	19.7	19.7	20.3	0.22
Current smoker (men)^c^	74.5	75.1	74.2	0.76
Current drinker (men)^c^	35.7	35.5	34.9	0.32
Fresh fruit >3 days per week^c^	27.7	27.4	27.7	0.87
Meat >3 days per week^c^	47.3	47.2	47.0	0.58
Dairy >3 days per week^c^	10.6	10.5	10.6	0.97
Systolic blood pressure (mmHg)^b^	128.9 (19.0)	129.0 (18.4)	128.6 (19.0)	0.21
Diastolic blood pressure (mmHg)^b^	77.0 (10.8)	77.1 (10.4)	77.6 (10.8)	<0.0001
Pulse pressure (mmHg)^b^	51.9 (13.2)	51.8 (12.8)	51.0 (13.2)	<0.0001
Mean arterial pressure (mmHg)^b^	94.3 (12.6)	94.4 (12.2)	94.6 (12.6)	<0.0001
Height (cm)^b^	158.6 (5.6)	158.8 (5.5)	158.9 (5.6)	<0.0001
Weight (kg)^b^	59.3 (9.4)	59.5 (9.1)	59.6 (9.4)	<0.0001
Body mass index (kg/m^2^)^b^	23.5 (3.3)	23.5 (3.2)	23.5 (3.3)	0.15
Body fat (%)^b^	27.9 (6.7)	27.9 (6.5)	27.8 (6.7)	0.78
Physical activity (MET-h/day)^b^	22.7 (12.8)	22.8 (12.4)	22.8 (12.8)	0.15
Random blood glucose (mmol/L)^b^^,^^d^	5.9 (2.1)	5.9 (2.0)	5.9 (2.1)	0.01

a All analyses are stratified by region and adjusted for age, age-squared, sex and regional principal components. CC, CT and TT refer to two cytosine, one cytosine and one thymine, and two thymine alleles at position 677 of the *MTHFR* polymorphism, respectively. Physical activity was quantified as metabolic equivalents of task hours per day (MET-h/d) based on the type, frequency and duration of specific activities.

b Estimates are adjusted means (standard error).

c Estimates are adjusted prevalences.

d There were missing values for random blood glucose by MTHFR genotypes. There were 38 406, 50 883 and 22 982 participants with random plasma glucose data for CC, CT and TT, respectively.

**Figure 1.**
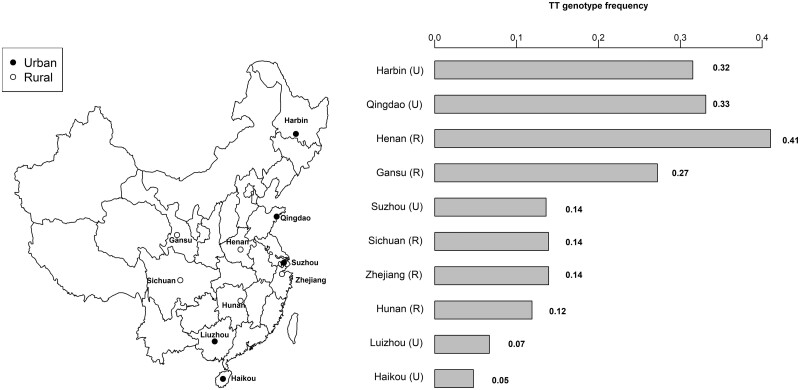
Locations of sites and frequency of the *MTHFR* C677T TT genotype by study area. TT refers to two thymine alleles at position 677 of the *MTHFR* polymorphism

Overall, 21% of participants were homozygotes for the TT genotype, with an 8-fold difference in TT prevalence between the Northern and Southern regions in China (Figure 1). Across the 10 study regions, the prevalence of TT genotype varied from 41% in Henan to 5% in Haikou (Figure 1). Across 10 study regions, there was no evidence of departure from HWE in seven regions (Supplementary Table S4, available as Supplementary data at *IJE* online) but there was evidence of departure from HWE in Liuzhou and minimal departure in Sichuan and Zheijang.

### Associations of *MTHFR* C677T with vascular disease outcomes

**Figure 2.**
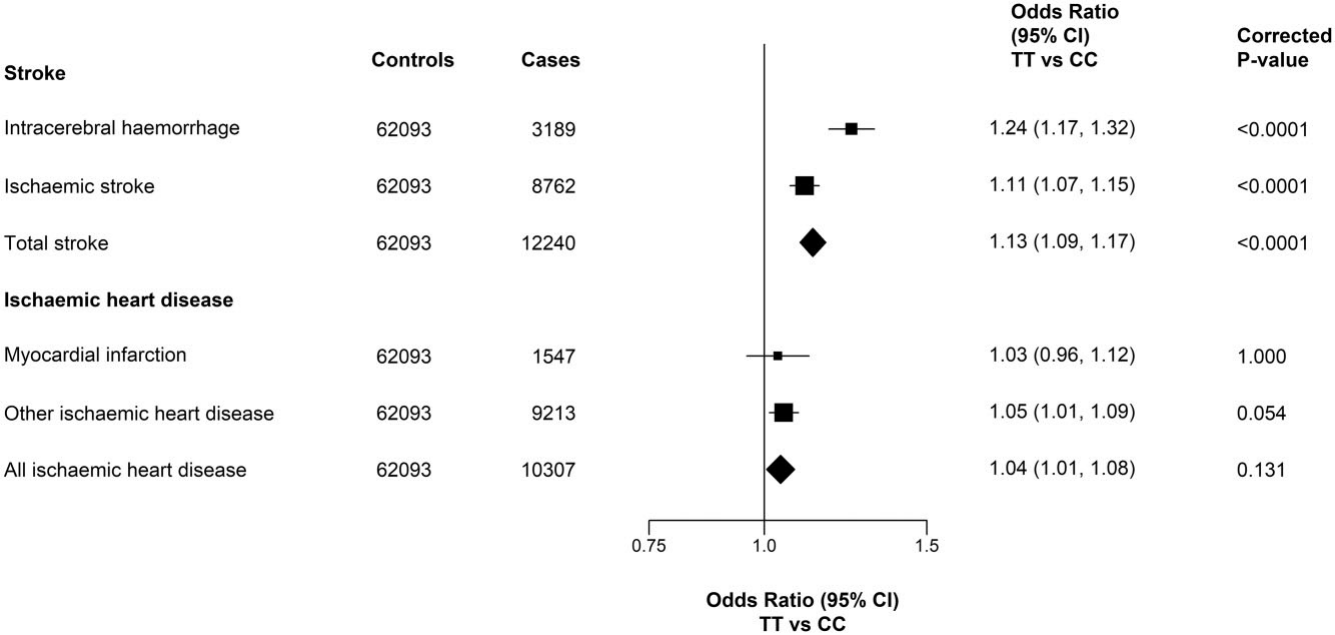
Association of *MTHFR* C677T with incident cases of stroke and ischaemic heart disease. Values shown are the odds ratios (95% CIs). The sizes of the squares are proportional to the inverse variance of each effect size. All analyses are region stratified and adjusted for age, age-squared, sex and regional principal components. TT and CC refer to two thymine alleles and two cytosine alleles, respectively, at position 677 of the *MTHFR* polymorphism

Figure 2 shows the region stratified and adjusted results for all primary vascular disease outcomes. Individuals with TT genotypes for *MTHFR* C677T had 13% (adjusted OR = 1.13, 95% CI 1.09–1.17) higher risks of any stroke, with a 2-fold stronger association with intracerebral haemorrhage (*n* = 3189 cases; 1.24, 1.17–1.32) than for IS (*n* = 8762; 1.11, 1.07–1.15). In contrast, the *MTHFR* C677T polymorphism was unrelated to MI (1.03; 0.96–1.12), other IHD (1.05; 1.01–1.09) or all IHD (1.04; 1.00–1.08) outcomes after accounting for multiple testing (Figure 2).

Supplementary Figure S3 (available as Supplementary data at *IJE* online) assesses the impact of additional adjustment for different blood pressure indices over and above adjustment for age, sex, region and regional PCs. Adjustment for DBP attenuated the associations of *MTHFR* with total stroke (1.13 vs 1.09 for TT vs CC) and likewise for mean arterial pressure (1.13 vs 1.11) whereas adjustment for pulse pressure inflated the associations with total stroke (1.13 vs 1.16, respectively). The patterns were similar for both IS and ICH, whereby adjustment for DBP attenuated ORs but pulse pressure increased the ORs and similarly for IHD.

Supplementary Figure S4 (available as Supplementary data at *IJE* online) demonstrates the importance of appropriate adjustment for region and regional PCs because, in the absence of such adjustments, the ORs for stroke were much more extreme than those with such adjustments. For example, the ORs for total stroke declined after full adjustment from 1.55 (95% CI: 1.50–1.60) to 1.13 (1.09–1.17). Likewise, the ORs for MI without such adjustments was 1.51 (1.40–1.63), which was attenuated to 1.04 (0.96–1.13) after adjustment (Supplementary Figure S4, available as Supplementary data at *IJE* online), illustrating the substantial confounding by failure to take account of regional differences in TT prevalence. There was no evidence of heterogeneity in the strength of the associations of *MTHFR* C677T TT vs CC with ICH, IS or total stroke (Supplementary Figure S5, available as Supplementary data at *IJE* online) across the 10 study regions.

Table 2 shows the associations of all three *MTHFR* C677T genotypes (TT, CT and CC) with risks of incident vascular disease outcomes. Compared with CC, the heterozygote CT genotype (one cytosine and one thymine allele at position 677 for the *MTHFR* C677T polymorphism) had less than half the effect of the TT genotype on stroke risk, with ORs per T allele of 1.09 (1.04–1.13), 1.05 (1.02–1.07) and 1.05 (1.03–1.08) for ICH, IS and total stroke, respectively. In the analyses corresponding to a 25% higher tHcy, the adjusted ORs were 1.19 (1.10–1.28) for ICH, 1.10 (1.04–1.16) for IS and 1.12 (1.07–1.17) for total stroke, respectively (Table 2), consistently with the results for the TT vs CC genotypes in Figure 2. Likewise, there was no evidence of an association per T allele or per 25% higher tHcy with MI or total IHD after taking account of multiple testing. The effects of *MTHFR* C677T on stroke and IHD outcomes were largely unaltered in sensitivity analyses that excluded prior vascular disease in cases and controls, or when analyses were restricted to a random population subset with genome-wide genotyping data with additional exclusions of those recruited for nested case–control studies (Supplementary Table S5, available as Supplementary data at *IJE* online).

**Table 2.** Effects of *MTHFR* C677T genotype on incident vascular outcomes

Outcome	**Number of cases** ^a^			**Odds ratio (95% CI)** ^b^				
	CC	CT	TT	CC	CT	TT	Per T allele	**Per 25% higher tHcy** ^c^
Intracerebral haemorrhage	1805	2684	1384	1.00 (0.95–1.05)	1.09 (1.04–1.13)	1.24 (1.17–1.32)	1.09 (1.05–1.13)	1.19 (1.10–1.28)
Ischaemic stroke	4670	7220	4092	1.00 (0.97–1.03)	1.05 (1.02–1.07)	1.11 (1.07–1.15)	1.05 (1.02–1.08)	1.10 (1.04–1.16)
Total stroke	6658	10 142	5582	1.00 (0.97–1.03)	1.05 (1.03–1.08)	1.13 (1.09–1.17)	1.06 (1.03–1.08)	1.12 (1.07–1.17)
Myocardial infarction	846	1335	701	1.00 (0.93–1.07)	1.05 (0.99–1.11)	1.03 (0.96–1.12)	1.02 (0.96–1.08)	1.03 (0.92–1.14)
Other ischaemic heart disease	5103	7956	4110	1.00 (0.97–1.03)	1.03 (1.01–1.06)	1.05 (1.01–1.09)	1.00 (0.98–1.03)	1.00 (0.95–1.05)
All ischaemic heart disease	5671	8945	4636	1.00 (0.97–1.03)	1.03 (1.01–1.06)	1.04 (1.01–1.08)	1.01 (0.98–1.03)	1.01 (0.96–1.05)

a The cases are compared with a common set of controls for each outcome. The number of controls for each *MTHFR* C677T genotype was 38 908 for CC, 51 479 for CT and 23 185 for TT. CC, CT and TT refer to two cytosine, one cytosine and one thymine, and two thymine alleles at position 677 of the *MTHFR* polymorphism, respectively. tHcy refers to plasma total homocysteine levels.

b All analyses were region stratified and adjusted for age, age-squared and regional principal components.

c OR per 25 higher tHcy is the odds ratio weighted by the expected effects of *MTHFR* C677T genotypes on homocysteine levels: TT = 1, CT = 0.25, CC = 0.

The effects of *MTHFR* C677T on risk of total stroke were more extreme in younger than in older people and in individuals living in rural than in urban areas, but did not differ by sex, education, smoking, alcohol or diagnosed hypertension (Supplementary Figure S6, available as Supplementary data at *IJE* online). Moreover, there was no evidence of effect modification for stroke by the two alcohol genetic variants rs1229984 and rs671 (Supplementary Figure S7, available as Supplementary data at *IJE* online).

### Associations with non-vascular disease outcomes

**Figure 3.**
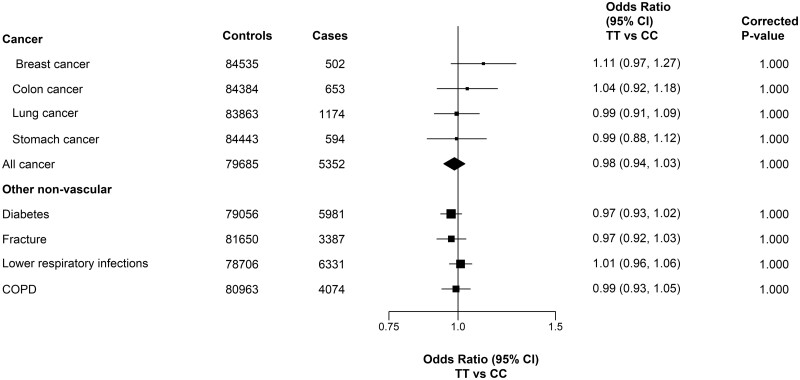
Association of *MTHFR* C677T with incident cases of cancer and other non-vascular diseases. Values shown are the odds ratios (95% CIs). The sizes of the squares are proportional to the inverse variance of each effect size. All analyses are region stratified and adjusted for age, age-squared, sex and regional principal components. TT and CC refer to two thymine alleles and two cytosine alleles, respectively, at position 677 of the *MTHFR* polymorphism. COPD, chronic obstructive pulmonary disease

There was no evidence of any association of *MTHFR* C677T genotypes with any of the pre-specified non-vascular disease outcomes in the adjusted models (Figure 3). In sensitivity analyses without stratification for 10 study regions or adjustment for PCs, there was substantial confounding that resulted in much more extreme ORs for non-vascular disease outcomes [e.g. ∼30% lower risks for lower respiratory tract infections, chronic obstructive pulmonary disease (COPD) and fracture, respectively: Supplementary Figure S8, available as Supplementary data at *IJE* online]. In a phenome-wide analysis of *MTHFR* C677T and 41 disease outcomes (Supplementary Figure S9, available as Supplementary data at *IJE* online), there were no associations of *MTHFR* C677T with any other diseases apart from stroke after correction for multiple testing.

## Discussion

This genetic study of 156 253 Chinese adults reported that the overall TT homozygote frequency for the *MTHFR C677T* polymorphism was 2-fold greater in Chinese than in Western populations but the TT frequency also differed by 8-fold between the 10 study regions (41% in North vs 5% in South) in China. Individuals with TT compared with CC genotypes for *MTHFR C677T* had a 13% higher risk of stroke (24% higher risk of ICH and 11% higher risk of IS) but had no excess risk of IHD or any of the other diseases studied.

In contrast with previous studies,^15–17^ this genetic study in a Chinese population that stratified the analyses by the 10 study regions and adjusted for regional PCs should have minimized any risk of population stratification. This study provides important new evidence for the population relevance of elevated tHcy levels for risk of stroke prevention (particularly for ICH, but also for IS). However, the present study provided no additional support for the relevance of elevated tHcy levels for risk of IHD or for cancer, diabetes or respiratory diseases.

Consistently with the discrepant findings of *MTHFR C677T* levels with stroke incidence in Chinese vs European populations, a meta-analysis of trials of folic acid reported conflicting results in populations with or without folic acid fortification.^13^^,^^14^Supplementary Figure S10 (and Supplementary Methods, available as Supplementary data at *IJE* online) includes results of a meta-analysis of randomized trials of folic acid and shows that folic acid supplements had no protective effects on total stroke in populations with mandatory or voluntary folic acid fortification but had a protective effect in populations without folic acid fortification. The findings also reflect the effects of the CSPPT trial, which reported that folic acid supplements (∼0.8 mg daily in 20 702 Chinese adults with hypertension but no prior history of CVD) reduced the risk of total stroke by 21%. In contrast with the present genetic study, the CSPPT trial reported that folic acid lowered IS [*n* = 515 by 24% (95% CI: 9 to 36%), *n* = 515 cases] but had no power to detect an effect on ICH (*n* = 120 outcomes) or on IHD (*n* = 49 outcomes).^14^ The discrepant results of folic acid trials for stroke prevention between Chinese and Western populations should prompt further trials of folic acid for stroke prevention in the Chinese population.^25^

The *MTHFR C677T* polymorphism results in elevated tHcy concentrations (Supplementary Figure S1, available as Supplementary data at *IJE* online) but the tHcy-elevating effects of this polymorphism are attenuated by folic acid fortification.^3^ The effects of the *MTHFR C677T* polymorphism are also highly sensitive to population stratification but the present report involved meta-analysis of region-specific analyses with additional adjustment for PCs to minimize such biases. Moreover, the concordant results of the sensitivity analyses that excluded prior disease in cases and controls or restricted analyses to random population subsets should mitigate other potential biases.

High alcohol intake and related alcoholic liver disease are associated with folate deficiency,^23^^,^^24^ which could possibly modify the associations of *MTHFR* C677T with risks of stroke and other diseases. However, we observed no effects of adjustment for reported alcohol consumption on risks of stroke whereas *MTHFR C677T* only had a modest effect on blood pressure measures (albeit with opposing effects on SBP and DBP), indicating that this variant may have minor pleiotropic effects on blood pressure. However, stepwise adjustment for SBP, DBP, pulse pressure and mean arterial pressure had only a modest effect on the excess risks of stroke associated with TT vs CC genotypes for *MTHFR C677T*.

Previous studies had reported conflicting results about the associations of elevated plasma tHcy levels with risk of diabetes, fracture or cancer of the stomach or colon.^26–29^ The present study involving a phenome-wide analysis of 41 disease outcomes found no evidence that *MTHFR* C677T was related to any other major non-CVD outcomes in Chinese adults.

Mandatory food fortification with folic acid has been widely implemented in the Americas and Australia, and voluntary fortification has been implemented in Europe but many low- and middle-income countries, including China, have not yet implemented any effective folic acid fortification.^30^^,^^31^ A randomized trial of two types of flour—one fortified with vs one without folic acid—conducted in 16 648 Chinese women reported that folic acid fortification reduced the incidence of neural tube defects by 69%.^32^^,^^33^ The present study suggests that mandatory folic acid fortification may be beneficial in China and the absolute benefits may be greater in the north of China, where stroke incidence rates are 10-fold higher and prevalence rates of *MTHFR* TT genotypes are 8-fold greater than in the south of China.

The chief strengths of the present study include a large number of well-characterized stroke and other disease outcomes, control of population stratification in a population without folic acid fortification and high prevalence of the *MTHFR* TT genotypes. However, the present study also had some limitations. First, no measurements of plasma folate or tHcy concentrations were available but previous surveys in China have reported population mean plasma folate concentrations that are less than one-third of those in populations with mandatory folic acid fortification.^34^ Second, the present study had limited power to detect the effects of the *MTHFR* C677T polymorphism on site-specific cancers or diabetes.

Consistently with the well-established beneficial effects of folic acid for prevention of neural tube defects,^34^^,^^35^ the present study provides important evidence for the relevance of the *MTHFR* polymorphism for stroke in Chinese adults. The findings should also prompt further trials of folic acid for stroke prevention in China and for effective mandatory folic acid fortification for stroke prevention in the Chinese population.^36^

## Ethics approval

Ethics approval was obtained from relevant local (Oxford Tropical Research Ethics Committee 025–04), national and international ethics committees, and all participants provided written informed consent.

## Supplementary Material

dyad147_Supplementary_Data

## Data Availability

The observational data that support the findings of this study are available to bona fide researchers on application under the China Kadoorie Biobank Open Access Data Policy (www.ckbiobank.org). Sharing of genotyping data is constrained by the Administrative Regulations on Human Genetic Resources of the People’s Republic of China. Access to these is available through collaboration with CKB researchers.
