# Supplementary material for: MTHFR and risk of stroke and heart disease in a low-folate population: a prospective study of 156 000 Chinese adults
Source: Int J Epidemiol. 2023 Oct 28;52(6):1862–9. doi: 10.1093/ije/dyad147 (PMC10749746; doi:10.1093/ije/dyad147)
Supplement: Supplementary file 1 [file dyad147_supplementary_data.docx]

**Supplementary material for** 

**“MTHFR and risk of stroke and heart disease in a low folate population: a prospective study in 156,000 adults in the China Kadoorie Biobank”**.

**Table of contents**

Members of the China Kadoorie Biobank Study Collaborative Group	2

Supplementary methods	3

Table S1: Description of China Kadoorie Biobank incident diseases and their associated ICD-10 codes	9

Table S2: Selected characteristics for all participants and in the genetic sub-study of the China Kadoorie Biobank (CKB) study	10

Table S3: Associations of MTHFR C677T genotypes with all participant characteristics in China Kadoorie Biobank	11

Table S4: Hardy-Weinberg Equilibrium by region for MTHFR C677T genotypes in controls	12

Table S5: Sensitivity analyses of the effects of MTHFR C677T genotype on primary outcomes in the China Kadoorie Biobank (CKB) study	13

Figure S1: Overview of folate, vitamin B6, and vitamin B12 in homocysteine metabolism	14

Figure S2: Genetic information used for the analysis of MTHFR C677T in the China Kadoorie Biobank Study	15

Figure S3: Genetic information used for the analysis of MTHFR C677T in the China Kadoorie Biobank Study	16

Figure S4: The association of MTHFR C677T with incident vascular outcomes after accounting for population stratification	17

Figure S5: Association of TT vs CC of MTHFR C677T with (A) intracerebral haemorrhage (B) ischaemic stroke  (C) total stroke ordered by frequency of TT genotype	18

Figure S6: Association of MTHFR C677T by selected baseline characteristics with (A) intracerebral haemorrhage  (B) ischaemic stroke (C) total stroke	19

Figure S7: The association of MTHFR C677T with incidence of stroke by (A) ADH1B SNP (rs1229984) genotypes and (B) ALDH2 SNP (rs671) genotypes	20

Figure S8: The association of MTHFR C677T with incident non−vascular outcomes after accounting for population stratification	21

Figure S9: Phenome−wide association analysis of MTHFR C677T with 41 disease endpoints	22

Figure S10: Meta−analysis of randomised trials of folic acid on stroke incidence, by folic acid fortification category in diverse populations	23

# Members of the China Kadoorie Biobank Study Collaborative Group

**International Steering Committee:** Junshi Chen, Zhengming Chen (PI), Robert Clarke, Rory Collins, Liming Li (PI), Chen Wang, Jun Lv, Richard Peto, Robin Walters.

**International Co-ordinating Centre, Oxford**: Daniel Avery, Maxim Barnard, Derrick Bennett, Ruth Boxall, Ka Hung Chan, Yiping Chen, Zhengming Chen, Johnathan Clarke; Robert Clarke, Huaidong Du, Ahmed Edris Mohamed, Hannah Fry, Simon Gilbert, Pek Kei Im, Andri Iona, Maria Kakkoura, Christiana Kartsonaki, Hubert Lam, Kuang Lin, James Liu, Mohsen Mazidi, Iona Millwood, Sam Morris, Qunhua Nie, Alfred Pozarickij, Paul Ryder, Saredo Said, Dan Schmidt, Becky Stevens, Iain Turnbull, Robin Walters, Baihan Wang, Lin Wang, Neil Wright, Ling Yang, Xiaoming Yang, Pang Yao. 

**National Co-ordinating Centre, Beijing:** Xiao Han, Can Hou, Qingmei Xia, Chao Liu, Jun Lv, Pei Pei, Dianjianyi Sun, Canqing Yu

**10 Regional Co-ordinating Centres:** Guangxi Provincial CDC: Naying Chen, Duo Liu, Zhenzhu Tang. Liuzhou CDC: Ningyu Chen, Qilian Jiang, Jian Lan, Mingqiang Li, Yun Liu, Fanwen Meng, Jinhuai Meng, Rong Pan, Yulu Qin, Ping Wang, Sisi Wang, Liuping Wei, Liyuan Zhou. Gansu Provincial CDC: Caixia Dong, Pengfei Ge, Xiaolan Ren. Maiji CDC: Zhongxiao Li, Enke Mao, Tao Wang, Hui Zhang, Xi Zhang. Hainan Provincial CDC: Jinyan Chen, Ximin Hu, Xiaohuan Wang. Meilan CDC: Zhendong Guo, Huimei Li, Yilei Li, Min Weng, Shukuan Wu. Heilongjiang Provincial CDC:  Shichun Yan, Mingyuan Zou, Xue Zhou. Nangang CDC: Ziyan Guo, Quan Kang, Yanjie Li, Bo Yu, Qinai Xu. Henan Provincial CDC: Liang Chang, Lei Fan, Shixian Feng, Ding Zhang, Gang Zhou. Huixian CDC: Yulian Gao, Tianyou He, Pan He, Chen Hu, Huarong Sun, Xukui Zhang. Hunan Provincial CDC: Biyun Chen, Zhongxi Fu, Yuelong Huang, Huilin Liu, Qiaohua Xu, Li Yin. Liuyang CDC: Huajun Long, Xin Xu, Hao Zhang, Libo Zhang. Jiangsu Provincial CDC: Jian Su, Ran Tao, Ming Wu, Jie Yang, Jinyi Zhou, Yonglin Zhou. Suzhou CDC: Yihe Hu, Yujie Hua, Jianrong Jin, Fang Liu, Jingchao Liu, Yan Lu, Liangcai Ma, Aiyu Tang, Jun Zhang. Qingdao CDC: Liang Cheng, Ranran Du, Ruqin Gao, Feifei Li, Shanpeng Li, Yongmei Liu, Feng Ning, Zengchang Pang, Xiaohui Sun, Xiaocao Tian, Shaojie Wang, Yaoming Zhai, Hua Zhang, Licang CDC: Wei Hou, Silu Lv, Junzheng Wang. Sichuan Provincial CDC: Xiaofang Chen, Xianping Wu, Ningmei Zhang, Xiaoyu Chang. Pengzhou CDC: Xiaofang Chen, Jianguo Li, Jiaqiu Liu, Guojin Luo, Qiang Sun, Xunfu Zhong. Zhejiang Provincial CDC: Weiwei Gong, Ruying Hu, Hao Wang,Meng Wang, Min Yu. Tongxiang CDC: Lingli Chen, Qijun Gu, Dongxia Pan，Chunmei Wang, Kaixu Xie, Xiaoyi Zhang. 

# Supplementary methods

*China Kadoorie Biobank study population*

All permanent residents aged 35-74 years without known major disabilities were invited to join the study, and 499,500 (28% of 1801,167 invitees) participated, plus a further 13,215 who were slightly outside the target age range [1,2]. Participants attended survey clinics where health workers administrated a laptop-based questionnaire recording details of socioeconomic status, medical history, smoking, drinking, diet, and physical activity. Physical measurements included height, weight, waist and hip circumference, bioimpedance, lung function, heart rate, and blood pressure. Non-fasting blood samples were collected for immediate measurement of blood glucose levels and the remaining plasma was stored long-term for future assays.

*Statistical methods to control for population stratification*

 Principal component analysis (PCA) of 76,719 unrelated CKB participants identified 11 PCs informative for CKB population structure. Consistent with findings from many previous studies, individuals formed discrete clusters correlated strongly with longitude and latitude, mostly reflecting the geographic origins of the individuals recruited in each region. For three regions, however, the clusters of participants were clearly offset on a plot of the first 2 PCs compared to their recruitment location. In each case the apparent discrepancies can be explained by known, relatively recent, major, historical population movements: large scale migration in the 16th and 17th centuries AD from Guangdong to Hainan island; repopulation of the Chengdu Basin in Sichuan in the late 17th and early 18th centuries, a large proportion of migrants coming from Huguang (now Hubei/Hunan); and settlement of largely-unpopulated Manchuria in the late 19th-early 20th century, with the majority of settlers originating from Shandong province [3]. Among participants with genome-wide data, PCA was conducted to generate region-specific PCs for use as covariates in association analyses (two to nine PCs per region). For regional PCs, participants identified as outliers in the PCA were excluded from the GWAS association analyses (chiefly from Liuzhou that included a high proportion with non-local ancestry reflecting population migration after 1950s). Among participants without GWAS data, regional PCs were imputed using the mean values of other participants from their recruitment clinic.

*Additional statistical methods*

Additional analyses included stepwise comparisons of incremental adjustments in the logistic regression models of: (i) by age, sex, region and regional principal components; (ii) additional adjustments for different blood pressure indices (systolic blood pressure [SBP], diastolic blood pressure [DBP], pulse pressure [PP], mean arterial blood pressure [MAP]) and (iii) other CVD risk factors (e.g. smoking, alcohol and body mass index [BMI]) to account for potential pleiotropy or confounding. The likelihood ratio χ^2^_1_ statistic was used to assess the impact of adjustment for confounders on the strength of the associations with disease outcomes. Subgroup analyses were performed to assess potential effect modification for the main stroke outcomes by sex, age group, area, education, smoking, alcohol drinking and hypertension. Subgroup analyses were also performed on two genetic variants influencing alcohol consumption to assess possible interactions with alcohol consumption on the associations of MTHFR with stroke. 

A phenome-wide analysis using incident disease outcomes (each with ≥500 events in the range ICD-10 A00-N99 which were grouped into 41 distinct ICD-10 categories (with no exclusions for prevalent diseases in respective controls) was conducted. Logistic regression was performed separately within each region and then combined by inverse-variance weighted meta-analysis. A Bonferroni p-value threshold of 0.05/41 = 0.0012 was used to assess statistical significance in order to account for multiple testing.

*Meta-analysis of randomised trials of folic acid for stroke prevention*

A previous systematic review of randomized trials of folic acid for prevention of stroke was updated to include any additional trials of folic acid for stroke prevention that were reported prior 2023 [4].  Randomized trials were eligible if:  (i) they involved a double-blind randomized comparison of B-vitamin supplements containing folic acid vs placebo for the prevention of vascular disease (irrespective of whether any other treatment was administered factorially); (ii) the relevant treatment arms differed only with respect to the intervention to lower homocysteine levels (i.e., they were unconfounded); and (iii) the trial involved at least 1000 participants for a scheduled treatment duration of at least 1 year [5-16]. Unpublished trials were sought through electronic searches and discussions with other experts in the field, but none was found. Strokes were the first occurrence of ischaemic, haemorrhagic, or unclassified stroke (but not just transient cerebral ischaemia). Comparisons were intention-to-treat, time-to-event analyses of first events of a particular type occurring during the scheduled treatment period among all patients allocated to folic acid vs all allocated to the control treatment. The log-rank observed minus expected (*o* − *e*) statistics and their variances (*v*) [7] from each trial were summed to produce, respectively, a grand total observed minus expected statistic (*G*) and its variance (*V*). The 1-step estimate of the logarithm of the event rate ratio is then *G* /*V* with variance 1/ *V* (and 95% CI [*G* /*V* ] ± [1.96/ *V*^1/2^]). For n trials, the χ^2^ statistic for heterogeneity with *n* − 1 degrees of freedom (χ^2^_n − 1_) is *S* − (*G*^2^/ *V*), where S is the sum over all the trials of (*o* − *e*)^2^/ *v*.  The results of these trials of folic acid supplementation on total stroke incidence were stratified by presence of food fortification with folic acid as either mandatory, voluntary or no folic acid fortification, respectively. 

**Supplementary references** 

1.	Chen Z, Lee L, Chen J, Collins R, Wu F, Guo Y, et al. Cohort profile: the Kadoorie Study of Chronic Disease in China (KSCDC). Int J Epidemiol. 2005;34:1243–49.

2.	Chen Z, Chen J, Collins R, Guo Y, Peto R, Wu F, et al. China Kadoorie Biobank of 0·5 million people: survey methods, baseline characteristics and long-term follow-up. Int J Epidemiol. 2011;40:1652–66.

3.	Walters R, Millwood I, Lin K, et al. Genotyping and population structure of the China Kadoorie Biobank. medRxiv 2022.05.02.22274487; doi: https://doi.org/10.1101/2022.05.02.22274487.

4.	 Clarke R, Halsey J, Lewington S, et al. Effects of lowering homocysteine levels with B vitamins on cardiovascular disease, cancer, and cause-specific mortality: Meta-analysis of 8 randomized trials involving 37 485 individuals. Arch Intern Med. 

5.	Toole JF, Malinow MR, Chambless LE, Spence JD, Pettigrew LC, Howard VJ, Sides EG, Wang CH, Stampfer M. Lowering homocysteine in patients with ischemic stroke to prevent recurrent stroke, myocardial infarction, and death: the Vitamin Intervention for Stroke Prevention (VISP) randomized controlled trial. JAMA, 2004; 291:565–575. 

6.	Jamison RL, Hartigan P, Kaufman JS, Goldfarb DS, Warren SR, Guarino PD, Gaziano JM. Effect of homocysteine lowering on mortality and vascular disease in advanced chronic kidney disease and end-stage renal disease: a randomized controlled trial. JAMA 2007, 298: 1163-70.

7.	Albert CM, Cook NR, Gaziano JM, Zaharris E, MacFadyen J, Danielson E, Buring JE, Manson JE. Effect of folic acid and B vitamins on risk of cardiovascular events and total mortality among women at high risk for cardiovascular disease: a randomized trial. JAMA. 2008; 299:2027–2036. 

8.	Lonn E, Yusuf S, Arnold MJ, Sheridan P, Pogue J, Micks M, McQueen MJ, Probstfield J, Fodor G, Held C, Genest J. Homocysteine lowering with folic acid and B vitamins in vascular disease. N Engl J Med, 2006; 354:1567–1577. 

9.	VITATOPS Trial Study Group. B vitamins in patients with recent transient ischaemic attack or stroke in the VITAmins TO Prevent Stroke (VITATOPS) trial: a randomised, double-blind, parallel, placebo-controlled trial. Lancet Neurology, 2010 1;9(9):855- 65.18705976.

10.	Bostom AG, Carpenter MA, Hunsicker L, Jacques PF, Kusek JW, Levey AS, McKenney JL, Mercier RY, Pfeffer MA, Selhub J, FAVORIT Study Investigators. Baseline characteristics of participants in the Folic Acid for Vascular Outcome Reduction in Transplantation (FAVORIT) trial. Am J Kidney Diseases, 2009 Jan 1;53(1):121-8. 

11.	Galan P, Kesse-Guyot E, Czernichow S, Briancon S, Blacher J, Hercberg S. Effects of B vitamins and omega 3 fatty acids on cardiovascular diseases: a randomised placebo controlled trial. BMJ, 2010; 341:c6273.

12.	Armitage JM, Bowman L, Clarke RJ, Wallendszus K, Bulbulia R, Rahimi K, Haynes R, Parish S, Sleight P, Peto R, Collins R. Effects of homocysteine-lowering with folic acid plus vitamin b12 vs placebo on mortality and major morbidity in myocardial infarction survivors: a randomized trial. JAMA, 2010; 303:2486–2494.

13.	van Wijngaarden JP, Swart KM, Enneman AW, Dhonukshe-Rutten RA, van Dijk SC, Ham AC, Brouwer-Brolsma EM, van der Zwaluw NL, Sohl E, van Meurs JB, Zillikens MC. Effect of daily vitamin B-12 and folic acid supplementation on fracture incidence in elderly individuals with an elevated plasma homocysteine concentration: BPROOF, a randomized controlled trial. Am J Clin Nutr, 2014; 100: 1578-86. 

14.	Bonaa KH, Njolstad I, Ueland PM, Schirmer H, Tverdal A, Steigen T, Wang H, Nordrehaug JE, Arnesen E, Rasmussen K. Homocysteine lowering and cardiovascular events after acute myocardial infarction. N Engl J Med, 2006; 354:1578–1588.

15.	Ebbing M, Bleie O, Ueland PM, Nordrehaug JE, Nilsen DW, Vollset SE, Refsum H, Pedersen EK, Nygard O. Mortality and cardiovascular events in patients treated with homocysteine-lowering B vitamins after coronary angiography: a randomized controlled trial. JAMA, 2008; 300:795–804. 

16.	Huo Y, Li J, Qin X, et al. Efficacy of Folic Acid Therapy in Primary Prevention of Stroke Among Adults With Hypertension in China: The CSPPT Randomized Clinical Trial. JAMA, 2015; 313:1325–1335.

17.	Yusuf S, Peto R, Lewis J, Collins R, Sleight P. Beta blockade during and after myocardial infarction: An overview of the randomized trials. Progress in Cardiovascular Disease 1985; 27: 335–371.

# Table S1: Description of China Kadoorie Biobank incident diseases and their associated ICD-10 codes

| **Incident disease** | **ICD-10 codes** |
| --- | --- |
| Ischemic stroke | I63 |
| Intracerebral hemorrhage | I61 |
| All stroke | I60-I61, I63-I64 |
| Acute myocardial infarction | I21-I23 |
| Other acute ischemic heart disease | I24 |
| All ischemic heart disease | I20-I25 |
| Breast cancer | C50 |
| Stomach cancer | C16 |
| Colon cancer | C18-C20 |
| Lung cancer | C33-C34 |
| All cancers | C00-C97 |
| Type II diabetes | E11-E14 |
| Fracture | S02-S92, M80 |
| Lower respiratory infections | J20-J22 |
| Chronic obstructive pulmonary disease | J41-J44 |

# Table S2: Selected characteristics for all participants and in the genetic sub-study of the China Kadoorie Biobank (CKB) study

| **Characteristic** | **All participants** **in the CKB study** **(n = 512,726)** | **Participants in** **the genetic** **sub-study** **(n = 156253)** |
| --- | --- | --- |
| **Demographic** |  |  |
| Age, years | 52.0 (10.7) | 52.4 (10.8) |
| Sex, %female | 302522 (59.0%) | 91803 (58.9%) |
| **Prior disease** |  |  |
| Heart disease (%) | 15472 (3.0%) | 4789 (3.1%) |
| Stroke/TIA (%) | 8884 (1.7%) | 2551 (1.6%) |
| Hypertension (%) | 59703 (11.6%) | 19195 (12.3%) |
| Diabetes (%) | 30301 (5.9%) | 9675 (6.2%) |
| Cancer (%) | 2578 (0.5%) | 703 (0.5%) |
| **Cardiovascular risk factors** |  |  |
| BMI, kg/m^2^ | 23.7 (3.4) | 23.7 (3.4) |
| Hip circumference, mm | 909.3 (68.7) | 908.7 (69.9) |
| Waist circumference, mm | 802.8 (97.6) | 802.5 (98.3) |
| Waist-hip ratio | 0.9 (0.1) | 0.9 (0.1) |
| Random blood glucose, mmol/L | 6.1 (2.3) | 6.1 (2.4) |
| SBP, mmHg | 131.1 (21.3) | 132.4 (22.0) |
| DBP, mmHg | 77.8 (11.2) | 78.3 (11.5) |
| Physical activity, MET-h/day | 21.1 (13.9) | 20.9 (14.0) |
| Current regular smoker (%) | 166084 (32.4%) | 51317 (32.9%) |
| Current regular drinker (%) | 76144 (14.9%) | 23561 (15.1%) |
| **Medication use** |  |  |
| Statin use (%) | 1145 (0.2%) | 355 (0.2%) |
| Aspirin use (%) | 5380 (1.0%) | 1788 (1.1%) |
| Blood pressure lowering (%) | 14405 (2.8%) | 4626 (3.0%) |
| Values are mean (SD) unless otherwise stated. All values are adjusted for age, sex and region where appropriate. There were 346 participants with missing *MTHFR* C677T data. |  |  |

# Table S3: Associations of MTHFR C677T genotypes with all participant characteristics in China Kadoorie Biobank

| **Participant characteristics** | **MTHFR genotypes** |  |  | **P-value** **for trend** |
| --- | --- | --- | --- | --- |
|  | **CC** **(n = 51581)** | **CT** **(n = 70896)** | **TT** **(n = 33456)** |  |
| Age (years) ^†^ | 52.4 (11.1) | 52.5 (10.7) | 52.4 (11.1) | 0.56 |
| % Female ^**ǂ**^ | 59.0 | 59.0 | 58.4 | 0.22 |
| High school education or above ^**ǂ**^ | 19.6 | 19.6 | 20.0 | 0.26 |
| Current smoker ^**ǂ**^ | 32.7 | 33.0 | 33.2 | 0.12 |
| Current drinker ^**ǂ**^ | 15.1 | 15.1 | 15.1 | 0.88 |
| Fresh fruit > 3 days per week ^**ǂ**^ | 27.5 | 27.6 | 27.8 | 0.43 |
| Meat > 3 days per week ^ǂ^ | 46.2 | 46.1 | 45.9 | 0.58 |
| Dairy > 3 days per week ^ǂ^ | 12.0 | 12.1 | 12.0 | 0.97 |
| Systolic blood pressure (mmHg) ^†^ | 132.3 (21.1) | 132.5 (20.4) | 132.2 (21.1) | 0.63 |
| Diastolic blood pressure (mmHg) ^†^ | 78.0 (11.6) | 78.2 (11.2) | 78.7 (11.6) | <0.0001 |
| Pulse pressure (mmHg) ^†^ | 54.3 (14.6) | 54.3 (14.1) | 53.5 (14.6) | <0.0001 |
| Mean arterial pressure (mmHg) ^†^ | 96.1 (13.8) | 96.3 (13.3) | 96.5 (13.8) | <0.0001 |
| Height (cm) ^†^ | 158.5 (5.7) | 158.6 (5.5) | 158.8 (5.7) | <0.0001 |
| Weight (kg) ^†^ | 59.6 (9.6) | 59.8 (9.3) | 59.9 (9.6) | <0.0001 |
| Body mass index (kg/m^2^) ^†^ | 23.6 (3.4) | 23.7 (3.3) | 23.7 (3.4) | 0.06 |
| Body fat (%)^†^ | 27.9 (6.8) | 28.0 (6.6) | 27.9 (6.8) | 0.93 |
| Physical activity (MET-h per day) ^†^ | 20.9 (12.4) | 20.9 (11.9) | 20.9 (12.4) | 0.56 |
| Random blood glucose (mmol per L) ^†^ ^§^ | 6.1 (2.5) | 6.1 (2.4) | 6.1 (2.5) | 0.10 |

^*^All analyses are stratified by region and adjusted for age, age-squared, sex, and regional principal components

†Estimates are adjusted means (standard error).

^**ǂ**^ Estimates are adjusted prevalences.

§There were missing values for random blood glucose by MTHFR genotypes. **There were 50936, 70130, 33194 participants with random plasma glucose data for CC, CT and TT respectively.**

# Table S4: Hardy-Weinberg Equilibrium by region for MTHFR C677T genotypes in controls  

|  | ***MTHFR*** **C677T genotype in controls** |  |  | **Hardy-Weinberg Equilibrium Chi-square (1 df)** | **Hardy-Weinberg Equilibrium p-value** |
| --- | --- | --- | --- | --- | --- |
| **Region** | **CC** | **CT** | **TT** |  |  |
| Qingdao (urban) | 1569 | 4172 | 2942 | 1.756 | 0.185 |
| Harbin (urban) | 1783 | 4578 | 3139 | 2.433 | 0.119 |
| Haikou (urban) | 3463 | 1747 | 235 | 0.555 | 0.456 |
| Suzhou (urban) | 5061 | 5896 | 1751 | 0.245 | 0.618 |
| Liuzhou (urban) | 5468 | 3320 | 610 | 11.911 | 0.00056 |
| Sichuan (rural) | 5545 | 6449 | 2048 | 5.970 | 0.015 |
| Gansu (rural) | 2874 | 6265 | 3486 | 0.324 | 0.569 |
| Henan (rural) | 1736 | 6102 | 5412 | 0.054 | 0.817 |
| Zhejiang (rural) | 5976 | 7397 | 2125 | 4.632 | 0.031 |
| Hunan (rural) | 5433 | 5553 | 1437 | 0.097 | 0.763 |
| **All regions** | **38908** | **51479** | **23185** | **651.311** | **1.159e-143** |

# Table S5: Sensitivity analyses of the effects of MTHFR C677T genotype on primary outcomes in the China Kadoorie Biobank (CKB) study

| **Outcome** | **Main results** |  | **Excluding prior disease in cases and controls** |  | **GWAS population sample** **excluding prior disease** **in cases and controls** |  |
| --- | --- | --- | --- | --- | --- | --- |
|  | **Cases*** | **Odds ratio (95%CI)** | **Cases*** | **Odds ratio (95%CI)** | **Cases*** | **Odds ratio (95%CI)** |
| Intracerebral haemorrhage | 3189 | 1.24 (1.17, 1.32) | 3017 | 1.25 (1.18, 1.33) | 1002 | 1.32 (1.20, 1.45) |
| Ischaemic stroke | 8762 | 1.11 (1.07, 1.15) | 7741 | 1.10 (1.06, 1.14) | 6164 | 1.10 (1.05, 1.14) |
| Total stroke | 12240 | 1.13 (1.09, 1.17) | 11019 | 1.12 (1.08, 1.16) | 7324 | 1.11 (1.07, 1.16) |
| Myocardial infarction | 1547 | 1.03 (0.96, 1.12) | 1412 | 1.00 (0.92, 1.08) | 760 | 1.11 (0.99, 1.24) |
| Other ischaemic heart disease | 9213 | 1.05 (1.01, 1.09) | 7838 | 1.03 (0.99, 1.07) | 6389 | 1.03 (0.99, 1.08) |
| Ischaemic heart disease | 10307 | 1.04 (1.01, 1.08) | 8842 | 1.02 (0.99, 1.06) | 6900 | 1.03 (0.99, 1.07) |

^*^ The cases are compared to a common set of controls for each outcome. 

The number of controls for each *MTHFR* C677T genotypes were CC=38908, CT=51479, TT=23185

All analyses were region-stratified and adjusted for age, age-squared and regional principal components

# Figure S1: Overview of folate, vitamin B6, and vitamin B12 in homocysteine metabolism

Homocysteine is reconverted to methionine by receiving a methyl group from 5-methyltetrahydrofolate, the active form of folate, or betaine in the remethylation pathway. Irreversible removal of homocysteine occurs through the transsulphuration pathway where homocysteine condenses with serine to form cystathionine. (Adapted from Yuan et al 2021 with permission).

# Figure S2: Genetic information used for the analysis of MTHFR C677T in the China Kadoorie Biobank Study

IS: Ischemic stroke; ICH: Intracerebral haemorrhage; TS: Total stroke; MI: Myocardial infarction; IHD: Ischemic heart disease;

# Figure S3: Genetic information used for the analysis of MTHFR C677T in the China Kadoorie Biobank Study

PCs: Are regional principal components derived from GWAS data; df:degrees of freedom; Base model: stratified by region and adjusted for age ,sex age−squared, regional principal components

# Figure S4: The association of MTHFR C677T with incident vascular outcomes after accounting for population stratification

PCs: Are regional principal components derived from GWAS data; df:degrees of freedom; CVD risk factors: smoking, alcohol, body mass index, physical activity and fresh fruit intake

# Figure S5: Association of TT vs CC of MTHFR C677T with (A) intracerebral haemorrhage (B) ischaemic stroke 
(C) total stroke ordered by frequency of TT genotype

These analyses are region stratified and adjusted for age, age−squared, sex and regional principal components.

# Figure S6: Association of MTHFR C677T by selected baseline characteristics with (A) intracerebral haemorrhage 
(B) ischaemic stroke (C) total stroke

All analyses were stratified by region and adjusted for age, age−squared, sex and regional principal components.

# Figure S7: The association of MTHFR C677T with incidence of stroke by (A) ADH1B SNP (rs1229984) genotypes and (B) ALDH2 SNP (rs671) genotypes

All analyses are region stratified and adjusted for age, age−squared, sex and regional principal components. P−het: P−value for heterogeneity

# Figure S8: The association of MTHFR C677T with incident non−vascular outcomes after accounting for population stratification

PCs: Are regional principal components derived from GWAS data; df:degrees of freedom; *LRI: Lower respiratory infections; COPD: Chronic obstructive pulmonary disease; CVD risk factors: smoking, alcohol, body mass index, physical activity and fresh fruit intake

# Figure S9: Phenome−wide association analysis of MTHFR C677T with 41 disease endpoints

All analyses are stratified by region and adjusted for age, age−squared, sex and regional principal components.

# Figure S10: Meta−analysis of randomised trials of folic acid on stroke incidence, by folic acid fortification category in diverse populations
